# Highly Sensitive Micropatterned Interdigitated Electrodes for Enhancing the Concentration Effect Based on Dielectrophoresis

**DOI:** 10.3390/s19194152

**Published:** 2019-09-25

**Authors:** Hye Jin Kim, Heeju Ahn, David S. Lee, Dongsung Park, Jae Hyun Kim, Jinsik Kim, Dae Sung Yoon, Kyo Seon Hwang

**Affiliations:** 1 Department of Clinical Pharmacology and Therapeutics, College of Medicine, Kyung Hee University, Seoul 02453, Korea; hyejinkim.mail@gmail.com (H.J.K.); hjahn18@korea.ac.kr (H.A.); dslee91@outlook.com (D.S.L.); dpark7047@gmail.com (D.P.); jae0221@khu.ac.kr (J.H.K.); 2 Department of Biomedical Engineering Korea University, Seoul 01897, Korea; dsyoon@korea.ac.kr; 3 Department of Medical Biotechnology, Dongguk University, Seoul 04620, Korea; lookup2@dongguk.edu

**Keywords:** dielectrophoresis, concentration effect, high sensitivity, amyloid beta 42, tau-441

## Abstract

The concentration effect of dielectrophoresis (DEP) enables detection of biomolecules with high sensitivity. In this study, microstructures were patterned between the interdigitated microelectrodes (IMEs) to increase the concentration effect of DEP. The microstructures increased the electric field gradient ($\nabla \left|E^{2}\right|$) between the IMEs to approximately 6.61-fold higher than in the bare IMEs with a gap of 10 μm, resulting in a decreased optimal voltage to concentrate amyloid beta 42 (Aβ_42_, from 0.8 V_pp_ to 0.5 V_pp_) and tau-441 (from 0.9 V_pp_ to 0.6 V_pp_) between the IMEs. Due to the concentration effect of DEP, the impedance change in the optimal condition was higher than the values in the reference condition at 2.64-fold in Aβ_42_ detection and at 1.59-fold in tau-441 detection. This concentration effect of DEP was also verified by counting the number of gold (Au) particles which conjugated with the secondary antibody. Finally, an enhanced concentration effect in the patterned IMEs was verified by measuring the impedance change depending on the concentration of Aβ_42_ and tau-441. Our results suggest that microstructures increase the concentration effect of DEP, leading to enhanced sensitivity of the IMEs.

## 1. Introduction

Molecular analysis provides meaningful information such as food quality or safety in food sciences, degree of water or air pollution in environmental sciences, and presence of disease or therapeutic effect in life sciences [1,2,3]. In particular, analyses of biomolecules have continuously been desired since biomolecules are not only essential to one or more biological processes but also their deficiency or excess can cause various diseases. Biomolecules have various sizes ranging from sub-nanometer to several hundreds of micrometers, and are present in biological fluids such as blood, plasma, and cerebral spinal fluid (CSF) at extremely low concentrations [4,5]. Thus, to analyze the biomolecules for disease diagnosis, the ability to detect the biomolecules in biological fluids at high sensitivity is essential. General approaches for a highly sensitive analysis of biomolecules decrease the size of the analysis device into nanoscale or integrate various technologies into the device [6,7]. However, these approaches are sometimes challenging and inefficient as they are expensive and they require a complex fabrication process, as well as consideration of various unexpected variables in microscale or in a single device [8,9]. These limitations have garnered interest from the research community in various concentrating technologies such as ion concentration polarization (ICP) [10,11], electrophoresis (EP) [12], and dielectrohporesis (DEP) that can be integrated into the devices for a highly sensitive analysis [13,14,15,16]. Among these technologies, DEP has been widely used to concentrate the biomolecules due to its high selectivity and rapidity in manipulation.

DEP exerts a force on polarizable particles when the particles are subjected to a nonuniform electric field, resulting in manipulation of the particles [17,18,19]. Using force, the particles are placed on the reaction region, where the electric field forms ”dense” or ”loose”. The forces are defined as positive DEP (pDEP) force and negative DEP (nDEP) force, respectively. DEP force also influences the biomolecules by locating them to a specific region according to the density of the electric field, which is defined as the “concentration effect” [20,21]. DEP manipulates the biomolecules without a complex structure and process. Due to its simplicity and the concentration effect, various researchers have applied DEP in their devices for a highly sensitive analysis for biomolecules [22,23,24]. Chen et al. optically analyzed Shewanella oneidensis cells with real-time fluorescence imaging using the pDEP trapping technique and Nguyen et al. detected circulating tumor cells with a size of about tens of micrometer by combining pDEP manipulation and impedance measurement [22,23]. Additionally, Kim et al. applied the pDEP effect to the electrode for focusing and sensing the cell [24]. The aforementioned studies performed a highly sensitive analysis using the concentration effect of DEP force, but they were based on the optical analytical methods that require bulk equipment, or detected large biomolecules having a size of several micrometer or more such as bacteria and cell, or used the pDEP force having a low selectivity. Contrary to these studies, we have previously used the concentration effect of the nDEP force to manipulate the proteins and to electrically detect the manipulated proteins [21,25]. The intensity of the nDEP force was controlled by considering the size of the target molecules so that only the target molecules were placed on to the reaction region. Consequently, the nanosized target proteins were analyzed with high sensitivity and selectivity. However, the enhancing ratio of sensitivity by DEP force, which showed the concentration effect of DEP, was low at about 0.71.

In this study, microstructures were patterned between the interdigitated microelectrodes (IMEs) to increase to the concentration effect of DEP force, thereby allowing a highly sensitive analysis of the biomolecules. The microstructures were used as floating electrodes to increase the intensity of the electric field (E-field) between the electrodes, resulting in an increase of the concentration effect. The IMEs with microstructures, namely the patterned IMEs, were fabricated by microelectromechanical systems (MEMS) technology and were tested by detecting Aβ_42_ and tau-441, which are crucial markers for Alzheimer’s disease (AD). The electric field gradient ($\nabla \left|E^{2}\right|$) between the IMEs was verified with COMSOL Multiphysics and was compared to the value in the bare IMEs. The conditions of the optimal voltage to concentrate Aβ_42_ and tau-441 in the bare IMEs and patterned IMEs were verified by measuring the impedance change depending on the voltage. On the basis of their optimal voltage conditions, the concentration effect of DEP force on the surface of the bare IMEs and the patterned IMEs were demonstrated by counting the number of the gold (Au) nanoparticles conjugated with the secondary antibodies. Using DEP force, the number of the Au particles on the surface of the patterned IMEs was higher than that in the bare IMEs at approximately 2-fold in both Aβ_42_ and tau-441 detection. These results indicated that the concentration effect of DEP force was enhanced in the patterned IMEs. Moreover, enhancement of the sensitivities by the concentration effect of DEP force was verified in the bare and patterned IMEs, respectively, according to the concentration of Aβ_42_ and tau-441. Our results suggest that the patterned IMEs with a high concentration effect and voltage resolution for the DEP force can serve as a powerful platform for highly sensitive analysis.

## 2. Materials and Methods

### 2.1. Chemicals and Reagents

All chemicals used in the research were of research purity (99.999%) and used without further purifications. Sulfuric acid (H_2_SO_4_), hydrogen peroxide (H_2_O_2_), and isopropyl alcohol (IPA) were purchased from Daejung Chemical and Metals Co., Ltd. (Daejung, Korea) and 10 mM phosphate-buffered saline (PBS, pH 7.4, 15–20 mS/cm) and deionized water (D.W.) (18.2 MΩ·cm at 25 °C) were obtained from Corning Inc. (Corning, NY, USA).

Amyloid beta protein fragment 1–42 (Aβ_42_), 3-(ethoxydimethylsilyl)propylamine (APTMES), N-(3-dimethylaminopropyl)-N′-ethylcarbodiimide (EDC), and N-hydroxysuccinimide (NHs) were purchased from Sigma-Aldrich Inc. (St. Louis, MO, USA). Purified anti-β-amyloid, 1–16 (6E10) antibody and purified anti-β-amyloid, 1-42 (12F4) antibody were purchased from BioLegend Inc. (San Diego, CA, USA). Tau monoclonal antibody (tau-5) was purchased from Thermo Fisher Scientific Inc. (Waltham, MA, USA). Recombinant human tau-441 protein, anti-tau antibody (7B8), and gold conjugation kit (20 nm, 20 OD) were purchased from Abcam Plc. (Cambridge, UK).

Polydimethylsiloxane (PDMS, siloxane Sylgard® 184 silicone elastomer kit) was obtained from Dowhitech Silicone Co., Ltd. (Goyang, Korea).

### 2.2. Simulation to Calculate Profiles of the Electric Field Between the Electrodes

The intensity of the electric field between the IMEs was simulated by the finite element method (FEM) of COMSOL Multiphysics® (version 5.2 with electrostatic (es) model in AC/DC modules). The bare IMEs were composed of two electrodes with either a 3.5 μm or a 10 μm gap. The patterned IMEs were composed of the square pattern with the size of 3 μm between two electrodes with a gap of 10 μm. All IMEs were constructed on a silicon dioxide (SiO_2_) plane with a relative permittivity of 3.9 and were surrounded with 10 mM PBS with a relative permittivity of 80. The electrodes were composed of platinum with relative permittivity of 5. The detailed process is described in the Supplementary Information.

### 2.3. Fabrication of IMEs and Immobilization of Antibody

IMEs were fabricated through a microelectromechanical system (MEMS) process. An insulation layer of SiO_2_ with a thickness of 3000 Å was deposited on a 4 inch silicon (Si) wafer via thermal oxidation, followed by sputtering of tantalum and platinum (Ta/Pt) electrode layers with a thickness of 300 and 1500 Å. The electrode layer on the surface was then etched via photolithography (Figure S1).

The antibody was immobilized on the SiO_2_ surface using the previously described protocol for immunoassay [21]. First, the IMEs chip was cleaned with the piranha solution, a 4:1 (v/v) mixture of H_2_SO_4_ with H_2_O_2_, for 30 min to remove any organic or inorganic residues, rinsed with distilled water (DW) and with IPA. The cleaned IMEs surface was then activated with 1% (v/v) APTMES in IPA for 5 min at 80 °C, stabilized for 20 min at 37 °C, and sequentially rinsed with IPA, DW, and 10 mM PBS. Then, 10 μg/mL of antibody activated with 20 mM of EDC and 100 mM of NHs for 15 min at 37 °C was applied for immobilization on the SiO_2_ surface between the electrodes for 1 h. The primary antibodies used were 6E10 which is specific for the sequences 1–16 of Aβ [26], and tau-5 which is specific for the sequences 218–225 of tau [27]. After immobilization of the antibody, the IMEs chip and PDMS channel were bonded via Van der Waals force.

### 2.4. Sysnthesis of Au Labeled Secondary Antibody

The secondary antibodies, 12F4 and 7B8, were conjugated with the Au particles. The 12F4 is a mouse monoclonal antibody reactive to the C-terminus of Aβ_42_, specific for the isoform ending at the 42nd amino acid [28], and 7B8 is a mouse monoclonal antibody specific to amino acids 5–12 of tau protein with an immunogen of N-terminal tau peptide sequence. Each secondary antibody was labeled with Au particles using a gold conjugation kit and treated for 20 min to the reaction region where the reaction between the primary antibody and the target protein was completed. The concentration of Au conjugating antibodies treated on the surface was 0.175 mg/mL.

### 2.5. Image Measurement

The surfaces of the IMEs were analyzed using the scanning electrode microscopy (Nova Nano SEM 200, FEI Company, USA) with magnification scale at 25,000×. The SEM images were processed with Image J software (version 1.52a) to quantify the area covered by Au particles and the number of the particle on the reaction region of the IMEs.

### 2.6. Immunoassay and Signal Processing

Target molecules were detected by measuring the impedance signal of the IMEs in 1 mM PBS buffer. After the immobilization of the antibodies on the surface of the IMEs through the method described in Section 2.3, the initial impedance value of the IMEs was measured in the pure PBS buffer (recorded as “B”). Then, PBS solution with the target molecules was injected in to the PDMS channel to react with the immobilized antibody for 20 min (Figure S2). During the reaction, AC voltage was applied using a function generator (Arbitrary Waveform Generator, Rigol Technologies, Beijing, China). Afterwards, the IMEs were rinsed with pure PBS and the impedance value was measured (recorded as “A”). The impedance change resulting from the reaction between the antibody and the target molecules was verified as followed:

Impedance change [%] = (*A* − *B)* / *B* × 100 (1)

To increase the reliability of the results, only the data within the standard deviation range was used. Raw data below the ”noise level” and above the ”offset level” was filtrated out. The ”noise level” was calculated by averaging the impedance changes which occurred from the reaction between the IMEs without immobilized antibody and the solution without antigen (Figure S3). The ”offset level” was calculated by multiplying raw impedance change by 3, where raw impedance change was calculated by averaging the impedance changes caused by the antibody-antigen reaction, excluding the ”noise level”. Afterwards, the averaged value (Avg) and standard deviation (Std) for the filtered data were calculated. Based on these values, the data in the ranges below were obtained.

“Avg − Std” ≤ or ≤ “Avg + Std”(2)

The obtained data were further filtered out by repeating Equation (2). The final filtered data were the values of the impedance change that were used in this paper.

## 3. Results and Discussion

### 3.1. Enhancing the Intensity of the Electric Field

Intensity of the DEP force is related to the gradient of the square of the electric field intensity ($\nabla \left|E^{2}\right|$) as follows:

(3) $$F_{DEP}=\;2\pi r^{3}\varepsilon_{m}^{*}K\left(\omega \right)\nabla \left|E^{2}\right|$$

where r is the radius of the particle subjected to the non-uniform electric field and K(ω) is the Clausius–Mossoti factor subjected to the relation between the complex permittivity of the particle ($\varepsilon_{p}$) and the permittivity of the medium surrounding the particle ($\varepsilon_{m}^{*}$) [29]. The intensity of the electric field relates to the distance between the electrodes and the intensity of the voltage applied on the electrodes. A stronger electric field occurs in the IMEs with a small gap, than the IMEs with a large gap, however, when the distance between the electrodes was reduced, the antibody-antigen reaction region of the IMEs was reduced. Consequently, the sensitivity of the sensor was decreased.

**Figure 1:**
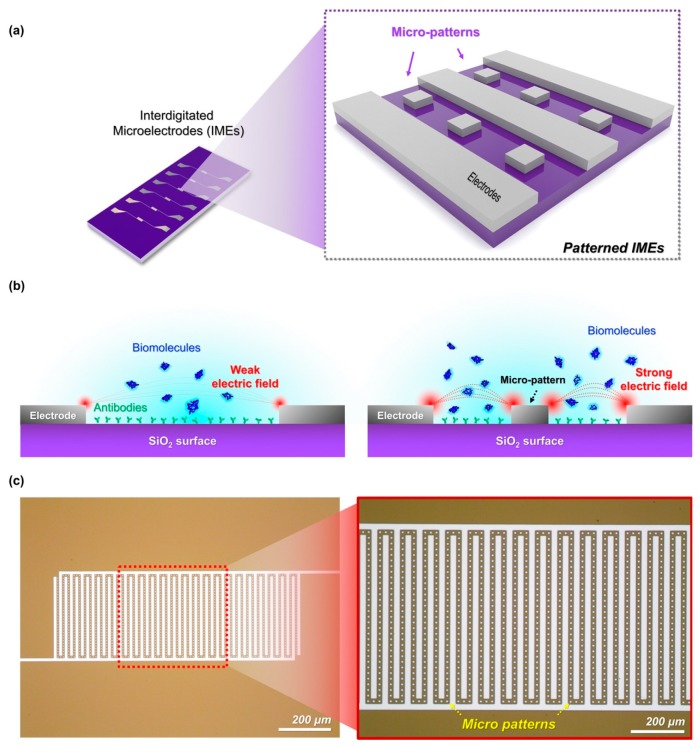
Highly sensitive electrical sensor with enhanced concentration effect of dielectrophoresis (DEP). (**a**) The patterned interdigitated microelectrodes (IMEs) have repetitive square microstructures between the IMEs, (**b**) reaction between the target molecules and their specific antibodies immobilized on the SiO_2_ surfaces of the bare IMEs (left) and the patterned IMEs (right), and (**c**) fabricated IMEs with micropatterns using MEMS technology.

In this study, IMEs with micropatterns were suggested for maximizing the intensity of the electric field of the same reaction region, as shown in Figure 1a. Unlike the general IMEs (bare IMEs, structure shown in Figure S1) which consist of a parallel pair of electrodes on the substrate, the patterned IMEs have arranged square micropatterns between the electrodes. As a result, the intensity of the DEP force in the patterned IMEs was relatively stronger than in the bare IMEs. The cross-sectional reaction between the antibody and the target molecules in each type of IMEs are shown in Figure 1b. It can be observed that when the target molecules reacted with the antibodies immobilized on the SiO_2_ surface (reaction region) of the bare IMEs, the molecules were influenced by a weak electric field resulting in a weak concentration on the reaction region. In contrast, the target molecules were highly concentrated on the reaction region of the patterned IMEs due to a strong electric field. The patterned IMEs after fabrication are shown in Figure 1c.

**Figure 2:**
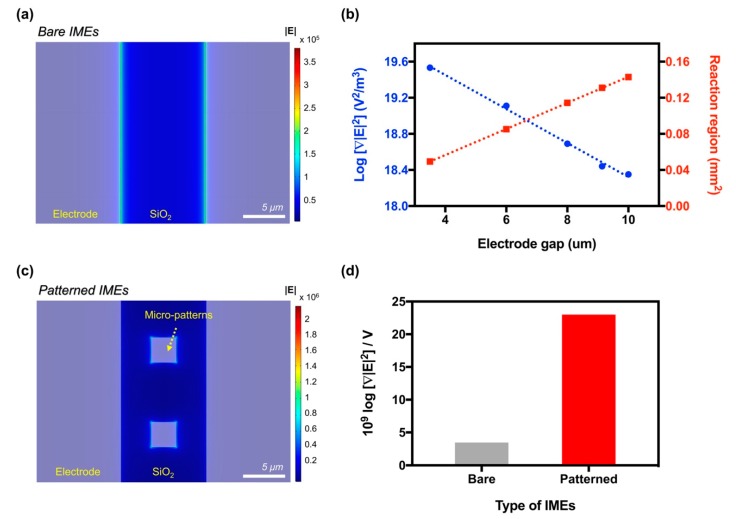
Enhancement of the electric field by the micropattern between the IMEs. (**a**) Intensity of the electric field on the surface of the bare IMEs when 0.5 V was applied, (**b**) intensity of the $\nabla \left|E^{2}\right|$ and the reaction region corresponding to the gap between the electrodes in the bare IMEs, (**c**) intensity of the electric field on the surface of the patterned IMEs when 0.5 V was applied, and (**d**) the value of the $\nabla \left|E^{2}\right|$ per voltage in the bare IMEs (gap: 10 μm) and in the patterned IMEs. The values were calculated through a power series fitting of the dots (y = Ax^B^ + Cx^D^) with 95% confidence.

When a voltage was applied on the bare IMEs, the strong electric field occurred at the edge of the electrodes and the electric field decreased depending on the distance from the edge, as shown in Figure 2a. The electric field gradient caused the DEP force between the electrodes, which concentrated the biomolecules in the area between the electrodes. The intensity of the electric field when the voltage of 0.5 V was applied on the bare IMEs was approximately 3.83 × 10^5^ V/m, which corresponded to the intensity of $\nabla \left|E^{2}\right|$ at approximately 2.25 × 10^18^ V^2^/m^3^. As shown in Figure 2b, the electric field gradient increased as the distance between the electrodes decreased. Approximately 3.41 × 10^19^ V^2^/m^3^ was observed in the IMEs with a gap of 3.5 μm, which was 15.16-fold higher than in the IMEs with a gap of 10 μm. However, the reaction region between the IMEs decreased concurrently from 0.14 mm^2^ to 0.05 mm^2^ as the electrode’s gap decreased. In contrast, the electric field in the patterned IMEs occurred at the edge of the electrodes as well as at the micropattern, as shown in Figure 2c. Thus, the intensity of $\nabla \left|E^{2}\right|$ was greater in the patterned IMEs than in the bare IMEs with the same electrode gap. Furthermore, the intensity in the patterned IMEs was greater than either values observed in the bare IMEs with a gap of 3.5 μm and 9.12 μm, where 3.5 μm was the distance between the pattern and the electrodes on patterned IMEs and 9.12 μm was the distance between the electrodes on bare IMEs, in order to match the total reaction region of the bare IMEs to that of the patterned IMEs. The intensity of $\nabla \left|E^{2}\right|$ was approximately 1.47 × 10^20^ V^2^/m^3^ in the patterned IMEs, 3.41 × 10^19^ V^2^/m^3^ in the bare IMEs with a 3.5 μm gap, and 2.76 × 10^18^ V^2^/m^3^ in the bare IMEs with a 9.12 μm gap (Figure S4). These results implied that the micropatterns effectively improved the intensity of the electric field while minimizing the reaction region loss. The increase in the electric field of the patterned IMEs implied that the patterned IMEs can cause the DEP force of the same intensity with comparably lower voltages than that of the bare IMEs. Through a power series fitting, $\nabla \left|E^{2}\right|$ per voltage was calculated to be approximately 2.30 × 10^10^ log$\nabla \left|E^{2}\right|$/V in the patterned IMEs, which was approximately 6.61-fold higher than in the bare IMEs (approximately 3.45 × 10^9^ log$\nabla \left|E^{2}\right|$/V). More detailed information for simulation were available in the Supplementary Information and Figure S5.

### 3.2. Optimizing the Intensity of the DEP Force

Aβ_42_ and tau-441 are crucial hallmarks involved in Alzheimer’s disease that are around 4.5 kDa and 45.8 kDa in size, respectively [30,31]. In order to concentrate Aβ42 and tau-441 to the reaction region, a frequency of AC voltage fixed at 50 MHz was applied to cause the negative DEP, which was the frequency that was optimized in our previous experiment [21]. Additionally, the conditions of voltage applied on the bare IMEs and patterned IMEs were optimized. Prior to the antigen reaction, antibodies were immobilized on the surface of the IMEs, as described in Section 2.3.

**Figure 3:**
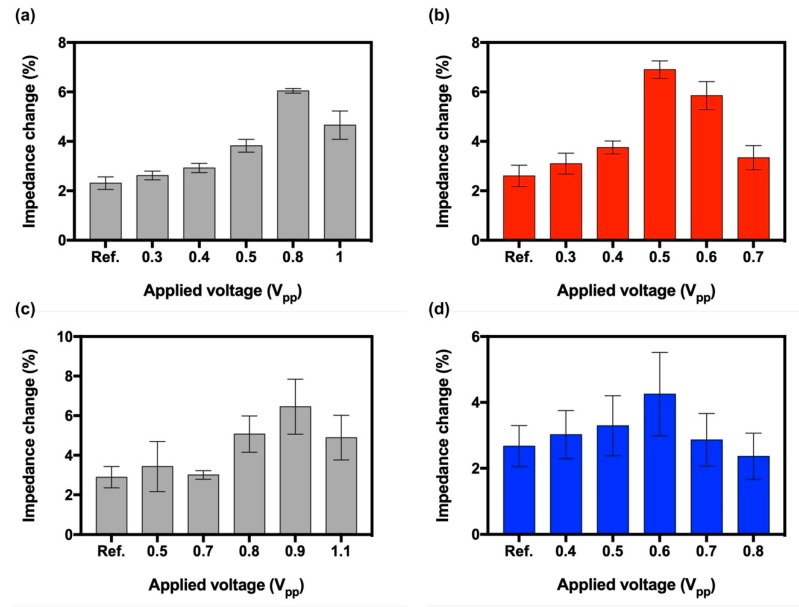
Optimization of the DEP condition in both patterned IMEs and bare IMEs. Impedance change by binding of Aβ_42_ in the (**a**) bare IMEs and (**b**) patterned IMEs; impedance change by binding of tau-441 in the (**c**) bare IMEs and (**d**) patterned IMEs, respectively. Error bars indicate standard deviations of 5 independent measurements at minimum.

In the bare IMEs, the impedance change from binding of 10 pg/mL of Aβ_42_ onto the 6E10 antibody was maximized to approximately 6.04 ± 0.09% when 0.8 V_pp_ was applied on the IMEs, as shown in Figure 3a. The impedance change in the bare IMEs was approximately 2.61-fold higher than the change when the intensity of the applied voltage was 0 V_pp_ (”Ref” in the Figure 3). For “Ref”, the impedance change was approximately 2.31 ± 0.25% and 2.61 ± 0.43% in the bare IMEs and the patterned IMEs, respectively. In the patterned IMEs, the impedance change increased to approximately 6.90 ± 0.36% when 0.5 V_pp_ was applied on the IMEs, as shown in Figure 3b. The ratio between these two impedance changes was approximately 2.64. As per the results, the optimal voltage condition (”DEP”) to concentrate Aβ_42_ was optimized to 0.8 V_pp_ for the patterned IMEs and 0.5 V_pp_ for the patterned IMEs.

The voltages of the bare and patterned IMEs were also optimized to concentrate the 10 pg/mL of tau-441, as shown in Figure 3c,b. In the bare IMEs, the impedance change was maximized at 0.9 V_pp_ to approximately 6.36 ± 1.38%, which was a 2.24-fold enhancement as compared to the value in ”Ref”, where the value was at about 2.89 ± 0.54%, however, the impedance change was maximized at 0.6 V_pp_ to approximately 4.25 ± 1.27%, which was about 1.59-times improvement over the impedance change in ”Ref”. These results suggest that the micropatterns constructed between the electrodes can increase the concentration of Aβ_42_ and tau-441 to the reaction region of the IMEs by applying the lower voltage than that required in the bare IMEs.

### 3.3. Verifying the Optimal DEP Force

**Figure 4:**
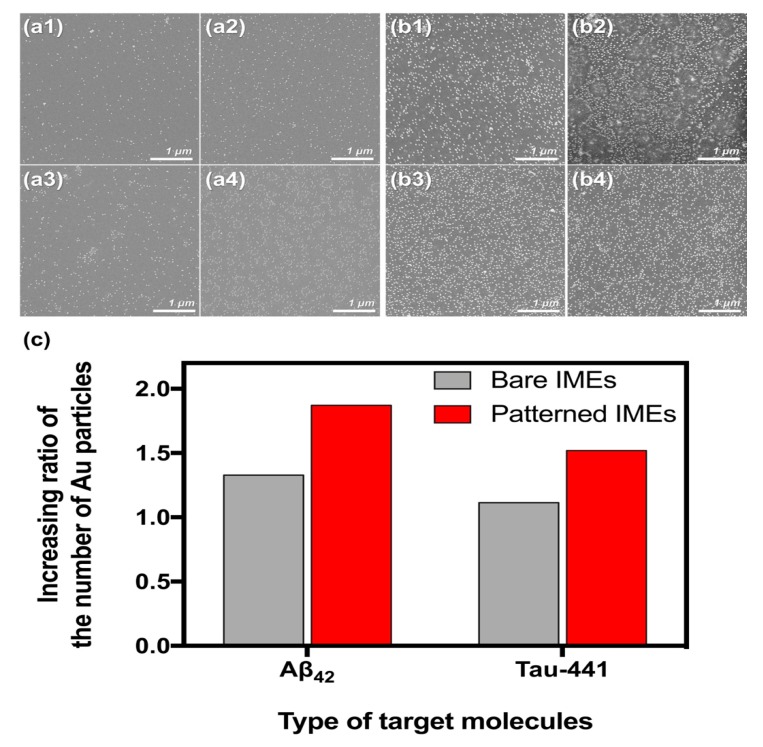
SEM images of the Au particles conjugated with secondary antibody on the surface of the IMEs: Bare IMEs surfaces after sequential treatment with (6E10)–(Aβ_42_)–(12F4) at *‘Ref.’* and at optimal voltage (0.8 V_pp_); with (tau-5)–(tau-441)–(7B8) at *‘Ref.’* and at optimal voltage (0.5 V_pp_) (**a1–a4**, respectively); patterned IMEs surfaces after sequential treatment with (6E10)–(Aβ_42_)–(12F4) at *‘Ref.’* and at optimal voltage (0.9 V_pp_); with (tau-5)–(tau-441)–(7B8) at *‘Ref.’* and at optimal voltage (0.6 V_pp_) (**b1–b4**, respectively); (**c**) Increasing ratio of the number of Au particles by the DEP force in the bare and patterned IMEs.

Concentrations of Aβ_42_ and tau-441 by the optimal DEP force were measured by counting the number of Au particles labeled with secondary antibody, as described in Section 2.4. The surfaces after the reaction between Aβ_42_ and its secondary antibody (12F4) were as follows (shown in Figure 4a): a1 and a2 are the surfaces of the bare IMEs in ”Ref” and optimal DEP condition at 0.8 V_pp_, and a3 and a4 are the surfaces of the patterned IMEs in the “Ref” and in the optimal DEP condition at 0.5 V_pp_, respectively. Additionally, the surfaces after the reaction between tau-441 and its secondary antibody (7B8) were as follows (shown in Figure 4b): b1 and b2 are the surfaces of the bare IMEs in ”Ref” and optimal DEP condition at 0.9 V_pp_, and b3 and b4 are the surfaces of the patterned IMEs in ”Ref” and optimal DEP condition (0.6 V_pp_), respectively.

To verify the concentration effect of DEP force, the number of the Au particles observed in ”Ref” was divided by the number of the particles observed in the optimal DEP condition. The calculated value was represented as an increasing ratio on the y-axis, as shown in Figure 4c. The increasing ratios of Aβ_42_ and tau-441 detections were approximately 1.33 and 1.11 in the bare IMEs and approximately 1.87 and 1.52 in the patterned IMEs, respectively. These results suggested that (1) Aβ_42_ and tau-441 were effectively concentrated on the reaction regions of the bare and patterned IMEs by the DEP conditions optimized in the Section 3.2; and (2) the concentration effect of DEP, the force was approximately 1.2-times greater in the patterned IMEs than in the bare IMEs.

### 3.4. Enhancing the Sensitivity in the Patterned IMEs

**Figure 5:**
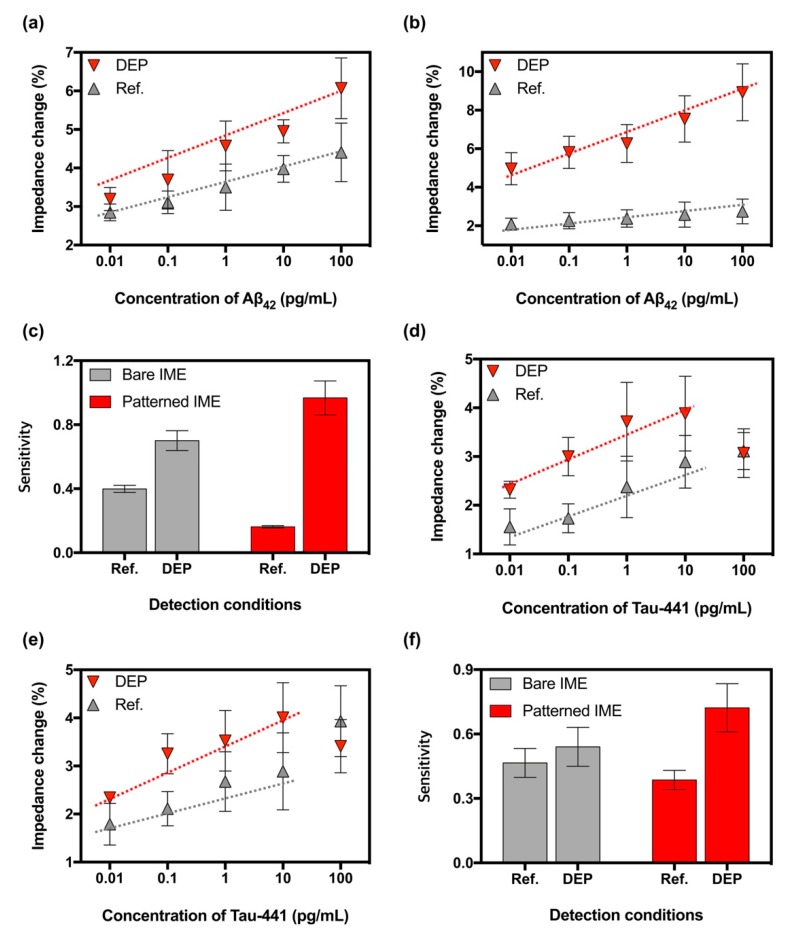
Impedance changes measured in (**a**) the bare IMEs and (**b**) the patterned IMEs, respectively, depending on various concentrations of Aβ_42_ in “DEP” (red) and “Ref” (gray). Dotted lines represent a regression with linearity to estimate the sensitivity. Error bars indicate standard deviations from minimum of 5 independent measurements. (**c**) Sensitivities calculated by linear regression with 95% confidence of the dotted lines in the bare IMEs (gray) and the patterned IMEs (red). Error bars indicate the standard errors. In addition, impedance changes measured in (**d**) the bare IMEs and (**e**) the patterned IMEs depending on the various concentration of tau-441 in the condition of “DEP” (red) and “Ref” (gray). Dotted lines represent a regression with linearity to estimate the sensitivity. Error bars indicate standard deviations from minimum of 5 independent measurements. (**f**) Sensitivities calculated by linear regression with 95% confidence of the dotted lines in the bare IMEs (gray) and the patterned IMEs (red), respectively. Error bars indicate the standard errors.

Impedance change and sensitivity were demonstrated by detecting various concentrations of Aβ_42_ and tau-441 in both the bare and the patterned IMEs. As shown in Figure 5a, the impedance changes by binding of Aβ_42_ in the bare IMEs ranged from approximately 2.85 ± 0.22% at 0.01 pg/mL to 4.41 ± 0.76% at 100 pg/mL in ”Ref”. The concentration effect of DEP force enhanced the impedance changes, which ranged from approximately 3.19 ± 0.30% at 0.01 pg/mL to 6.07 ± 0.79% at 100 pg/mL. Additionally, the impedance changes by in the patterned IMEs were observed, as shown in Figure 5b, which ranged from approximately 2.09 ± 0.31% at 0.01 pg/mL to 2.74 ± 0.64% at 100 pg/mL and 4.96 ± 0.83% at 0.01 pg/mL to 8.93 ± 1.48% at 100 pg/mL in ”Ref” and in ”DEP”, respectively.

Under these ranges, the sensitivities of the bare IMEs were calculated as a slope of the linear fitting curve, as shown in Figure 5c, around 0.40 ± 0.02 in “Ref” and 0.70 ± 0.06 in “DEP”, whereas those of the patterned IMEs were approximately 0.16 ± 0.01 in “Ref” and 0.97 ± 0.11 in “DEP”. Although the sensitivity of the patterned IMEs was less than that of the bare IMEs in “Ref” due to the decrease in the reaction region (from 0.1438 mm^2^ to 0.1310 mm^2^), the enhancement of the sensitivity by the concentration effect of DEP force in the patterned IMEs was greater than those of the bare IMEs. The enhancing ratios of sensitivities were approximately 1.75 in the bare IMEs and 6.06 in the patterned IMEs.

Additionally, impedance changes were measured according to the concentration of tau-441 in both the bare IMEs and the patterned IMEs to verify the enhancement of the sensitivity by the concentration effect of DEP force. Similar to the results from Aβ_42_ detection, the DEP force enhanced impedance changes in both IMEs. The changes measured in the bare IMEs ranged from approximately 1.56 ± 0.37% at 0.01 pg/mL to 3.11 ± 0.38% at 100 pg/mL in “Ref” and from 2.32 ± 0.17% at 0.01 pg/mL to 3.07 ± 0.50% at 100 pg/mL in ”DEP”, as shown in Figure 5d. Furthermore, the impedance changes in the patterned IMEs were approximately 1.79 ± 0.43% at 0.01 pg/mL to 3.93 ± 0.74% at 100 pg/mL in “Ref” and 2.34 ± 0.06% at 0.01 pg/mL to 3.41 ± 0.55% at 100 pg/mL in ”DEP”, as shown in Figure 5e.

On the basis of these results, the sensitivities were calculated to be approximately 0.47 ± 0.07 and 0.54 ± 0.09 in “Ref” and in “DEP”, respectively, and those of the patterned IMEs were approximately 0.39 ± 0.05 and 0.72 ± 0.11 in “Ref” and in “DEP”, respectively, as shown in Figure 5f. Similar to the results of the Aβ_42_ detection, the sensitivity of the patterned IMEs in “Ref” was lower than that of the bare IMEs due to smaller reaction region of the patterned IMEs as compared to the bare IMEs, however, the enhancing ratio of the sensitivities by the concentration effect of DEP force was at approximately 1.87, which was greater than that of the bare IMEs at approximately 1.16. Overall, the microstructures enhanced the detection sensitivity of the IME by 346% in Aβ_42_ and 161% in tau-441.

## 4. Conclusions

In this study, we suggest a refined method to improve the concentration effect of DEP by patterning microstructures in the IMEs and to verify the enhanced concentration effect using specific antibody-antigen reactions with two different biomolecules. The microstructures enhanced the electric field gradient, leading to enhancement of the concentration effect of DEP for the biomolecules, as well as increasing the sensitivity of the sensor. The microstructures enhanced the detection sensitivity of the IMEs by approximately 346% of Aβ_42_ and 161% of tau-441, respectively. The results indicate that the sensitivity of sensor was enhanced by maximizing the concentration effect of DEP. Our results show the potential of simply and accurately blood-based Alzheimer’s disease diagnosis based on the detection of Aβ_42_ and tau-441 protein.

## Supplementary Materials

The following are available online at https://www.mdpi.com/1424-8220/19/19/4152/s1, Figure S1: Structure of bare IMEs, Figure S2: Impedance change by specific binding of 10 pg/mL amyloid beta according to the applied time of DEP force, Figure S3: Impedance changes by adsorption of Aβ_42_ (red) and tau-441 (blue), Figure S4: COMSOL Multiphysics in the bare IMEs with 3.5 μm gap. (**a**) Intensity of the electric field on the surface when 0.5 V was applied and (**b**) intensity of the $\nabla \left|E^{2}\right|$ corresponding to the intensity of the electric field, Figure S5: 3D simulation of $\nabla \left|E^{2}\right|$ around the electrodes of (**a**) bare IMEs and (**b**) patterned IMEs.
