# Supplementary material for: Highly Sensitive Micropatterned Interdigitated Electrodes for Enhancing the Concentration Effect Based on Dielectrophoresis
Source: Sensors (Basel). 2019 Sep 25;19(19):4152. doi: 10.3390/s19194152 (PMC6806168; doi:10.3390/s19194152)
Supplement: Supplementary file 1 [file sensors-19-04152-s001.pdf]

Supplementary information

Simple structure of bare IMEs

The bare IMEs consists of a pair of IMEs. The bare IMEs were composed of two electrodes with a gap of 10 μm between the electrodes.

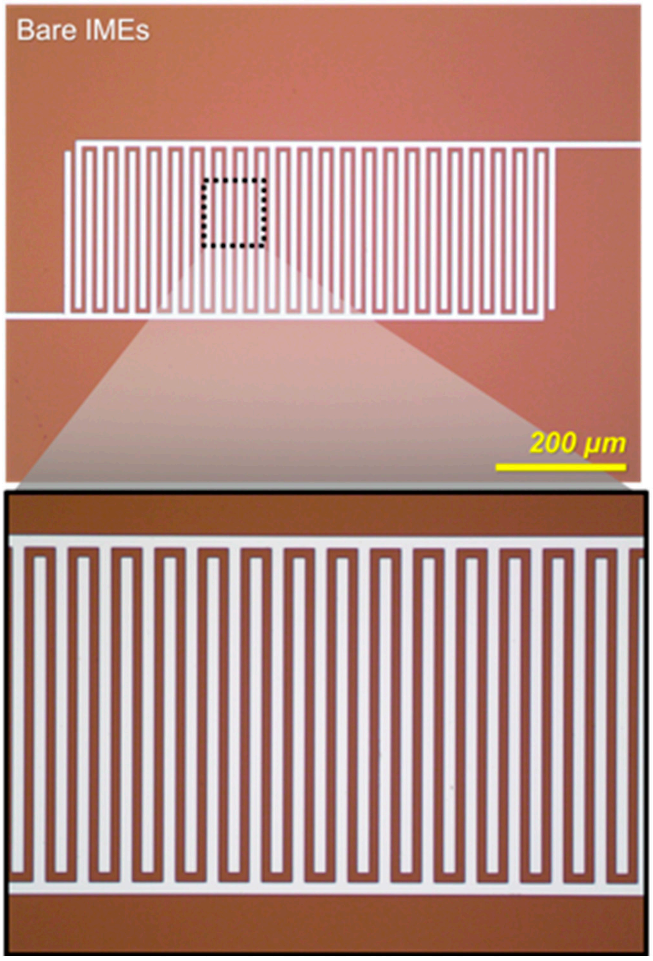

Figure S1. Structure of bare IMEs.

Impedance change according to the DEP time applied to the IMEs.

The impedance change according to the DEP time applied to the sensor was as followed:

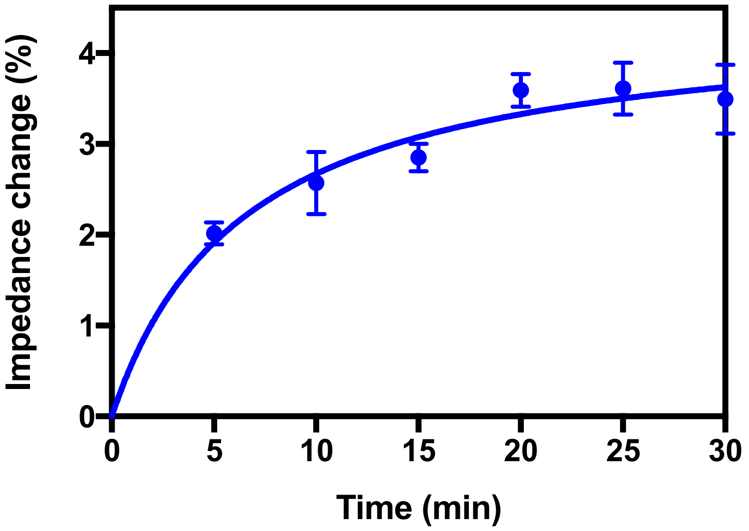

Figure S2. Impedance change by specific binding of 10 pg/mL amyloid beta according to the applied time of DEP force.

The impedance changes were observed every 5 min during 30 min reaction in the same IMEs. When the DEP force was applied to the IMEs for 5 min, the impedance change was approximately 2.015 ± 0.119% which saturated to 3.591 ± 0.182% after 20 min. Accordingly, the reaction time was adjusted to 20 min, considering that the efficiency of concentration induced by DEP forces was saturated after 20 min.

Verify the noise by adsorption of the biomolecules

To verify the noise level, Aβ_{42} and tau-441 with a concentration of 1 pg/mL each were adsorbed on the IMEs surface without the antibody immobilization process. As a result, the average impedance values (Avg.) with standard deviation (Std.) were approximately 1.21 ± 0.47 % in Aβ_{42} and 2.09 ± 0.51 % in tau-441.

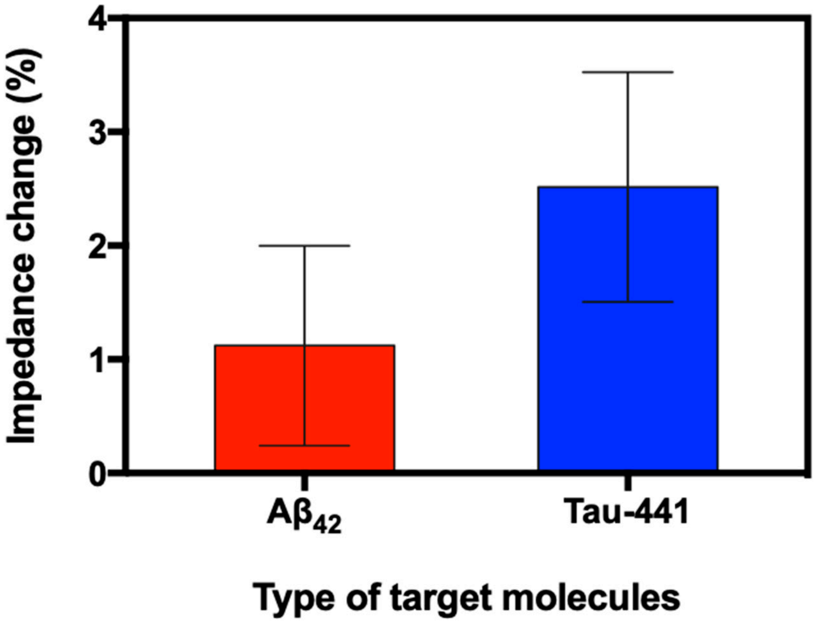

Figure S3. Impedance changes by adsorption of Aβ_{42} (red) and tau-441 (blue)

Simulation of the IMEs with small electrode gap

Electric field on the IMEs with a gap of 3.5 μm was simulated by COMSOL Multiphysics.

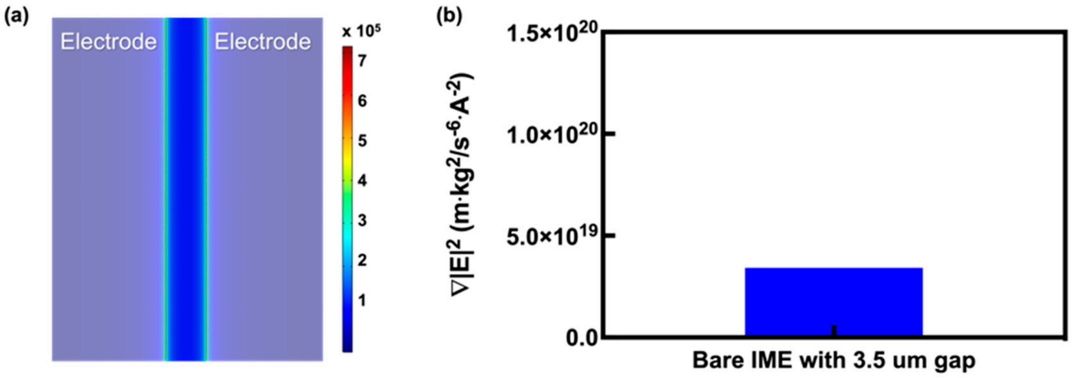

Figure S4. COMSOL Multiphysics in the bare IMEs with 3.5 μm gap. (a) Intensity of the electric field on the surface when 0.5 V_{pp} was applied; (b) Intensity of the $\nabla|E^2|$ corresponding to the intensity of the electric field.

Detailed information for COMSOL simulation

We simulated the IMEs through 3D modeling for accurate analysis, but the manuscript was submitted with a 2D simulated image in order to effectively represent the electric field on the IMEs surface. The 3D simulated images in the bare IMEs and patterned IMEs are shown in the Figure S5.

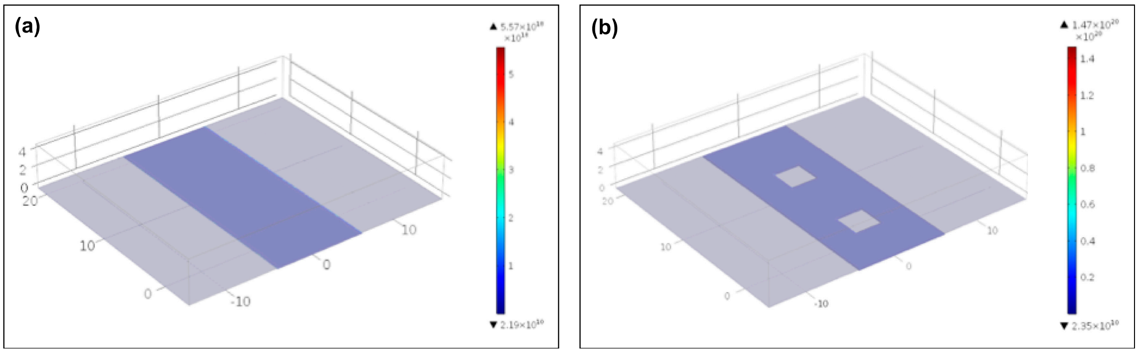

Figure S5. 3D simulation of $\nabla|\boldsymbol{E}|^2$ around the electrodes of (a) bare IMEs and (b) patterned IMEs.

For modeling of the bare IMEs, two platinum (Pt) electrodes that are 10 μm wide and 180 nm thick were constructed on a silicon dioxide (SiO_2) surface, as shown in Figure S5(a). Patterned IMEs were modeled on SiO_2 surface with two platinum electrodes having same dimensions as that of the bare IMEs and square patterns that are 3 μm wide and 180nm thick were placed between the electrodes, as shown in Figure S5(b). The electrodes were surrounded by PBS buffer, which was represented as a transparent cube.

We have used electrostatics model and calculated the value of electric field gradient ($\nabla|E|^2$) using equation as followed:

$$\nabla|E|^2 = \sqrt{d[(es.Ex)^2, x]^2 + d[(es.Ey)^2, y]^2 + d[(es.Ez)^2, z]^2}$$
